# A Biomimetic Plasmonic Nanoreactor for Reliable Metabolite Detection

**DOI:** 10.1002/advs.201903730

**Published:** 2020-03-11

**Authors:** Jiangang Liu, Chenlei Cai, Yuning Wang, Yu Liu, Lin Huang, Tongtong Tian, Yuanyuan Yao, Jia Wei, Ruoping Chen, Kun Zhang, Baohong Liu, Kun Qian

**Affiliations:** ^1^ Department of Neurosurgery Shanghai Children's Hospital Med‐X Research Institute and School of Biomedical Engineering Shanghai Jiao Tong University Shanghai 200062 China; ^2^ Department of Medical Oncology Shanghai Pulmonary Hospital Tongji University School of Medicine Shanghai 200433 China; ^3^ Department of Chemistry Institutes of Biomedical Sciences and State Key Lab of Molecular Engineering of Polymers Fudan University Shanghai 200438 China

**Keywords:** biomimetic nanoreactors, enzymes, macroporous silica foams, metabolic assays, metabolic testing, SERS biosensors

## Abstract

Reliable monitoring of metabolites in biofluids is critical for diagnosis, treatment, and long‐term management of various diseases. Although widely used, existing enzymatic metabolite assays face challenges in clinical practice primarily due to the susceptibility of enzyme activity to external conditions and the low sensitivity of sensing strategies. Inspired by the micro/nanoscale confined catalytic environment in living cells, the coencapsulation of oxidoreductase and metal nanoparticles within the nanopores of macroporous silica foams to fabricate all‐in‐one bio‐nanoreactors is reported herein for use in surface‐enhanced Raman scattering (SERS)‐based metabolic assays. The enhancement of catalytical activity and stability of enzyme against high temperatures, long‐time storage or proteolytic agents are demonstrated. The nanoreactors recognize and catalyze oxidation of the metabolite, and provide ratiometric SERS response in the presence of the enzymatic by‐product H_2_O_2_, enabling sensitive metabolite quantification in a “sample in and answer out” manner. The nanoreactor makes any oxidoreductase‐responsible metabolite a candidate for quantitative SERS sensing, as shown for glucose and lactate. Glucose levels of patients with bacterial infection are accurately analyzed with only 20 µL of cerebrospinal fluids, indicating the potential application of the nanoreactor in vitro clinical testing.

## Graphical abstract

Inspired by the spatially confined micro‐/nano‐environment in living cells, a plasmonic bio‐nanoreactor is developed by the coencapsulation of enzyme and surface‐enhanced Raman scattering tags within macroporous silica foams. The nanoreactor shows enhanced biocatalytic activity and stability, and thus the capability of molecular recognition and detection provides an all‐in‐one optical biosensor for reliable in vitro metabolic testing.

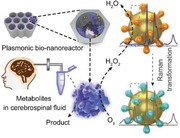

## 1 Introduction

The occurrence and progress of many diseases can result in abnormal metabolite flux in biofluids. For example, a malignant tumor or pathogenic bacterial infection status has been found to accompany with the sustained depletion of glucose and the concomitant elevation of lactate in the interstitial fluids due to the enhanced glycolysis energy metabolism.^[^ ^1^ ^]^ Accurate profiling of medically relevant metabolites in human body fluids provides molecular information for deep understanding of pathological mechanism at a more distal level than genomic analysis, and enables precise diagnosis and treatment of the underling diseases.^[^ ^2^ ^]^ Till now, different advanced techniques, including magnetic resonance spectroscopy,^[^ ^3^ ^]^ mass spectrometry,^[^ ^4^ ^]^ and chromatography coupled mass spectroscopy,^[^ ^5^ ^]^ have been developed to measure metabolites in vitro and in vivo. While useful, these techniques require sophisticated and expensive equipment, hampering their use in rapid point‐of‐care clinical testing. By contrast, enzyme‐based bioassays, where the presence of analytes is optically or electrochemically detected through the highly specific and efficient enzymatic reaction, provide novel opportunities for detection metabolite biomarkers.^[^ ^6^ ^]^ These approaches show enough figures of merits regarding device portability, speed, sample consumption and cost. However, the inherent instability of enzyme at elevated temperature, in circumstance containing digestive enzymes or chemical chelates, and when storing the enzyme long‐term represents a longstanding yet largely unsolved challenge that leads to poor reproducibility and accuracy in enzyme‐based metabolite analysis.^[^ ^7^ ^]^ Direct exposure to the external factors leads to changes in the enzyme structure or conformation, hence a decrease of the catalytic activity and fluctuation in the detection signal, which may result in ambiguities and even mistakes in sensing target metabolites,^[^ ^8^ ^]^ posing a difficulty in accurate diagnostics and evaluation of the therapy effectiveness.

Different from in vitro enzymatic reactions where the enzyme molecules are dissolved in a certain volume of buffer solution, biocatalytic transformations taking place in each living cell are controlled by enzymes within micro‐ and/or nanoscale confined environments. These surface‐ or volume‐confined enzymes are stable, efficient and capable of mediating reactions in a spatial defined manner.^[^ ^9^ ^]^ Inspired by such appealing features, substantial research efforts have been directed toward the fabrication of enzyme‐containing nanoreactors that mimic the discrete subcellular compartments. The encapsulation of enzymes in structures with nanoscale channels or pores provides a promising means to design biomimetic nanoreactors. Different approaches for spatially confining enzymes in micro‐ or nanoscale scaffolds have been reported, including the self‐assembly of enzymes into DNA origami nanocages,^[^ ^10^ ^]^ the encapsulation of enzymes in covalent organic framework,^[^ ^11^ ^]^ metal–organic framework,^[^ ^12^ ^]^ mesoporous silica,^[^ ^13^ ^]^ and carbon materials,^[^ ^14^ ^]^ etc. Our group has demonstrated the exploration of macroporous ordered silica foams (MOSF) incorporating enzymes as biomimetic nanoreactors to enhance proteolysis^[^ ^15^ ^]^ and functional proteomic identification in cancer cells,^[^ ^16^ ^]^ and to imitate in vitro drug metabolism.^[^ ^17^ ^]^ Similar to other 3D porous silica materials, MOSF shows typical features of large specific surface area, good stability, biocompatibility and water dispersibility, ease of preparation and surface functionalization, as well as light transparency. However, these nanoreactors are limited to qualitative analysis of biomacromolecules and are not compatible with in vitro quantitative determination of small molecule metabolites due to the lack of suitable signal conversion and readout ability.

Surface‐enhanced Raman scattering (SERS) is a powerful approach for measuring metabolites in biological fluids owing to its high sensitivity, nonbleaching, and fingerprinting natures.^[^ ^18^ ^]^ SERS sensors that function through enzyme‐based sensing (e.g., oxidoreductase‐mediated boronate oxidation), offer excellent selectivity toward specific metabolites, and have been used to profile metabolites of clinical importance (cholesterol, lactate, glucose, and H_2_O_2_, etc.) at the micromolar level.^[^ ^6c^ ^]^ Despite their utility, the previous SERS sensors execute their functions by enzymes dissolved in bulk solution, suffering from activity instability. A multifunctional nanoreactor that allows continuous robust enzymatic recognition and catalysis of target metabolites in complex biological samples, while working as a sensitive and user‐friendly optical biosensor, would be desirable and highly useful.

**Scheme 1:**
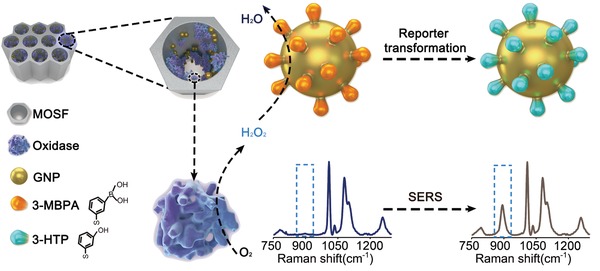
Schematic illustration of SERS‐based metabolite profiling with MOSF@GNPs@oxidases nanoreactors.

Against this backdrop, we reported the design and construction of a plasmonic bio‐nanoreactor by coimmobilizing boronate‐functionalized gold nanoparticles (GNPs) with oxidases on MOSF (denoted as MOSF@GNPs@oxidases). We first demonstrated that the oxidases confined in the nanopores of MOSF maintain catalytic activities over the free enzymes under harsh conditions. Besides, we verified the ability of the integrative nanoreactor to act as a smart metabolite sensing platform via enzymatic conversion of the analytes into H_2_O_2_, and hence the selective oxidation of the boronate probe into its corresponding phenol form, which can be spectrally detected using SERS in a ratiometric manner (**Scheme** **1**). In contrast to the conventional single intensity‐based detection that is easily affected by external factors, the ratiometric assay used here allows to eliminate such interferences by providing an internal spectral calibration.^[^ ^19^ ^]^ The nanoreactor is finally employed to measure the changes of cerebrospinal fluid (CSF) glucose associated with bacterial central nervous system (CNS) infections in postoperative neurosurgical patients, and to evaluate the clinical efficacy of anti‐infective therapy.

## 2 Results and Discussion

**Figure 1:**
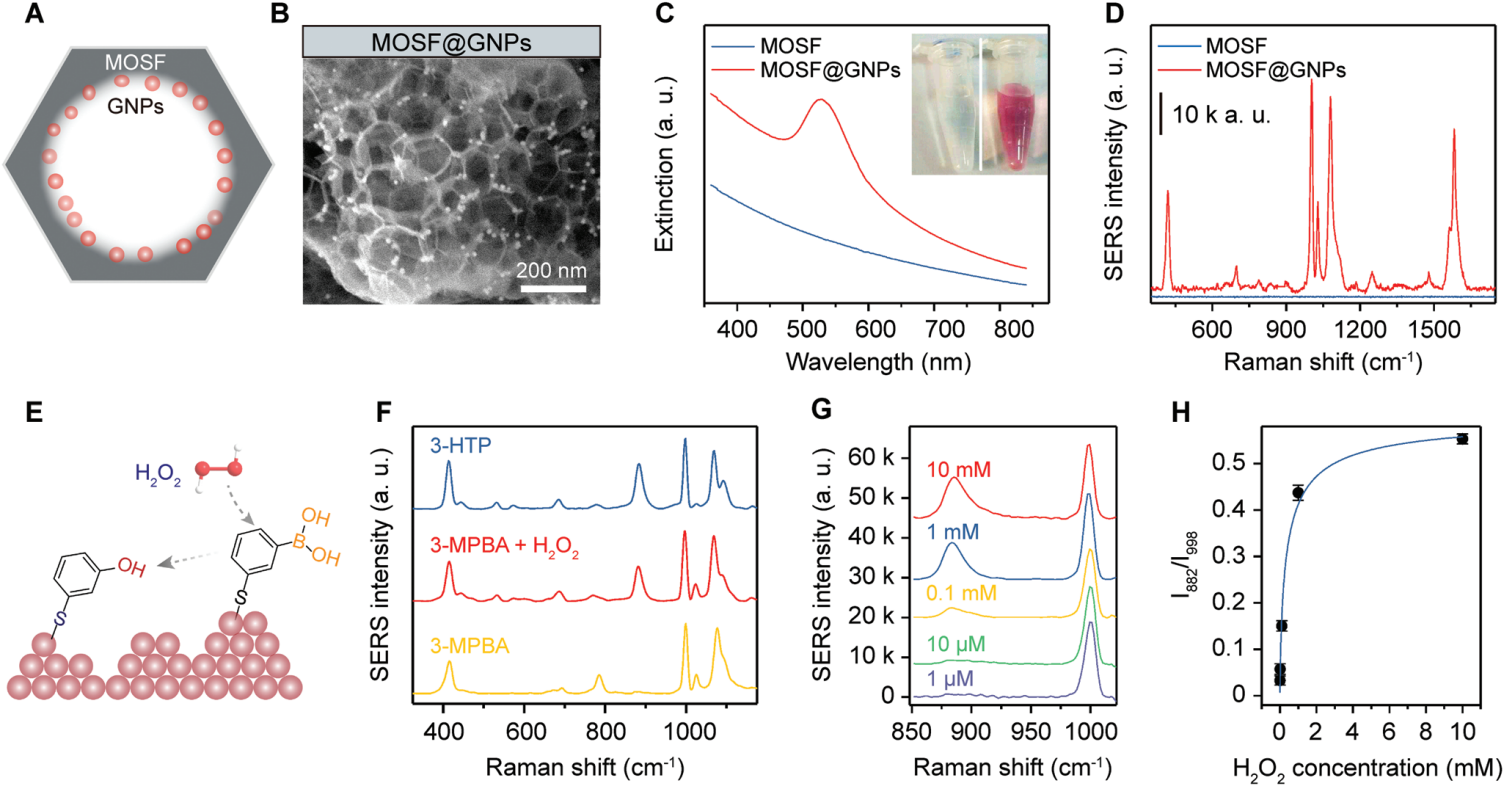
A) Scheme and B) SEM image of MOSF@GNPs. C) UV–vis extinction spectra of MOSF and MOSF@GNPs in water. D) SERS response of 3‐MPBA adsorbed on MOSF@GNPs. E) Scheme showing oxidation of 3‐MPBA to 3‐HTP by H_2_O_2_ at GNP surface. F) SERS spectra of 3‐HTP and 3‐MPBA before and after addition of H_2_O_2_. G) SERS spectra of 3‐MPBA recorded from surface of MOSF@GNPs as a function of H_2_O_2_ concentration. H) Peak intensity ratios (*I*_882_/*I*_998_) of the spectra shown in (G) as a function of H_2_O_2_ concentration. The error bars represent the standard deviation obtained from the mean of triplicate measurements.

MOSF materials were synthesized through an in‐solution assembling method by using P123 as the template and tetramethyl orthosilicate (TMOS) as the silica source.^[^ ^15^, ^16^ ^]^ Scanning electron microscopy (SEM) and transmission electron microscopy (TEM) characterizations indicated that the as‐prepared MOSF had macropores of ≈75 nm diameter with a wall thickness of ≈3–5 nm (Figure S1, Supporting Information). Nitrogen sorption analysis revealed that the silica foam had a surface area of 239 m^2^ g^−1^ and pore volume of 0.84 cm^3^ g^−1^, which was consistent with the microscopic analysis result (Figure S2, Supporting Information). To make this silica material SERS active, we first incubated it with GNPs. As expected, the metal nanoparticles were mainly adsorbed on the inner wall of the pores of MOSF (denoted as MOSF@GNPs, **Figure** **1**A,B). The nanohybrids were red in‐color and displayed a sharp peak at ≈528 nm in the UV–vis spectrum that was ascribed to the Mie absorption by the surface‐plasmon oscillation of spherical nanoparticles (Figure 1C).^[^ ^20^ ^]^ The red color combined with the single surface plasmon peak implied that the GNPs on MOSF were mainly located as isolated monomers. Notably, almost no GNPs were observed to fall off from the surface of MOSF during repeated washings, suggesting strong adsorption of the particles on MOSF (Figure S3, Supporting Information). Our previous studies indicated that MOSF materials were negatively charged in aqueous solution at near‐neutral pH.^[^ ^15a^ ^]^ Under this circumstance, GNPs also exhibit a negative zeta‐potential due to the surface‐adsorbed citrate (pKa 3.1, 4.8, and 6.4).^[^ ^21^ ^]^ Therefore, we excluded the possibility of electrostatic adsorption. The encapsulation of GNPs within MOSF might be attributed to the matched honeycomb‐like nanoporous structure of the silica foam, and the exact adsorption mechanism need to be further investigated.

To verify the SERS activity of MOSF@GNPs, we functionalized the GNP surface with a Raman reporter 3‐mercaptophenylboronic acid (3‐MPBA). Since spherical GNP monomers exhibit a relatively weak SERS activity,^[^ ^22^ ^]^ we pre‐concentrated the MOSF@GNPs before SERS measurements to increase the formation of electromagnetic hot spots. MOSF@GNPs dramatically enhanced the Raman signal of 3‐MPBA under optimized conditions (Figure 1D and Figures S4–S7, Supporting Information). The spectrum showed characteristic bands of 3‐MPBA at 415 (C–S stretching), 785 (C–H out‐of‐plane bending), 998 (C–C in‐plane bending), 1023 (C–H in‐plane bending), 1076 (C–C in‐plane bending coupled with C–S stretching), 1558 (nontotally symmetric ring stretching), and 1576 cm^−1^ (totally symmetric ring stretching).^[^ ^6^, ^18^ ^]^ After incubation in H_2_O_2_, the SERS spectrum of 3‐MPBA‐functionalized MOSF@GNPs was in good agreement with that of 3‐hydroxythiophenol (3‐HTP) (Figure 1F), indicating the transformation of boronate to phenol (Figure 1E). The new peaks at 882 and 1589 cm^−1^ were ascribed to ring stretching and totally symmetric ring stretching of 3‐HTP.^[^ ^6^, ^18^ ^]^ As depicted in Figure 1G, the band at 882 cm^−1^ increased gradually with increasing the H_2_O_2_ concentration, while the band at 998 cm^−1^ nearly unchanged. Therefore, we could detect H_2_O_2_ by analyzing the ratiometric peak intensity of *I*_882_/*I*_998_ against H_2_O_2_ concentrations, providing a linear dynamic range from 1 to 100 × 10^−6^ m with a theoretical limit of detection (LOD) of 0.47 × 10^−6^ m (Figure 1H). The MOSF@GNPs exhibited a comparable detection performance for H_2_O_2_ compared with previously reported SERS sensors (Table S1, Supporting Information).

**Figure 2:**
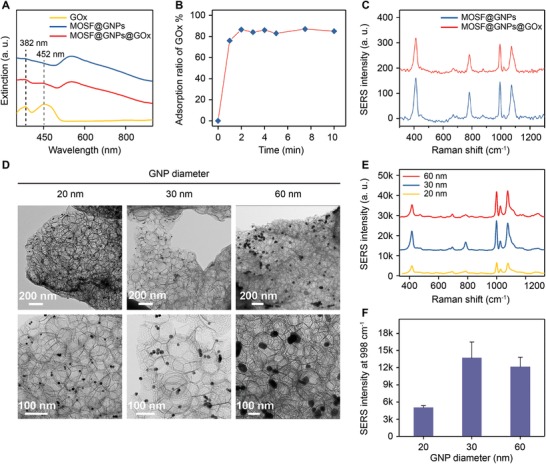
A) UV/vis extinction spectra of MOSF@GNPs before and after immobilization of GOx. B) Time‐dependent adsorption of GOx on MOSF@GNPs. C) SERS spectra of MOSF@GNPs before and after GOx immobilization. D) TEM images of MOSF@GNPs@GOx nanoreactors composed of 20, 30, and 60 nm GNPs. E) SERS spectra and F) 998 cm^−1^ peak intensity of 3‐MPBA obtained from MOSF@GNPs@GOx nanoreactors composed of different sized GNPs.

To enable accurate metabolic assay by the nanosensor, we further immobilized oxidoreductases in the MOSF@GNP nanocomposites. Glucose oxidase (GOx) was first used as a model enzyme. UV–vis spectra of the GOx aqueous solution showed two characteristic absorption bands at 382 and 452 nm (Figure 2A and Figure S8 in Supporting Information,). After incubation with the MOSF@GNP nanocomposite, the spectral intensity of the GOx solution decreased by approximately threefold. In addition, the two peaks of the enzyme appeared in the spectrum of the nanocomposite, suggesting the adsorption of GOx onto the porous nanocomposite. The absorbance measurement of the enzyme solution at different incubation intervals indicated that the maximum adsorption could be reached within 2 min, with a plateau adsorption capacity of ≈45 mg g^−1^ (**Figure** **2**B). This was consistent with our previous studies that MOSF showed a high affinity and fast loading kinetics for enzyme molecules even without additional surface functionalization.^[^ ^15^, ^16^, ^17^ ^]^ The SERS intensity of the nanocomposite reached 73.4% of its initial value after saturated GOx adsorption that was ascribed to the adsorption of the enzyme molecules on the gold surface (Figure 2C). Despite a slight decrease in the SERS intensity, the high signal‐to‐background ratio of the Raman spectrum demonstrated that enough SERS activity was remained after acquiring new functions of molecular recognition and catalytic transformation. To construct the optimized nanoreactors, we fabricated the nanoreactors with GNPs of different sizes (Figure 2D). As observed from Figure 2E,F, the SERS intensity of the nanoreactor increased by about 2.6 times when the GNP diameter increased from 20 to 30 nm. Further increase of the GNP size, however, resulted in no increase of the SERS intensity. This can be explained by the following aspects: first, the plasmonic activity of single spherical GNPs is positively correlated with the particle size;^[^ ^23^ ^]^ in addition, the total enhancement of a SERS substrate fabricated with the spherical GNPs is positively correlated with the surface coverage of the particles.^[^ ^24^ ^]^ The relatively high plasmonic activity of 30 nm GNPs combined with their large density in the MOSF nanopores provided the strong Raman response. Further, considering the surface property of MOSF, we believe that the number of GNPs in MOSF can be efficiently elevated by adjusting the surface charges or functional groups of MOSF (e.g., amino or thiol functionalization).

**Figure 3:**
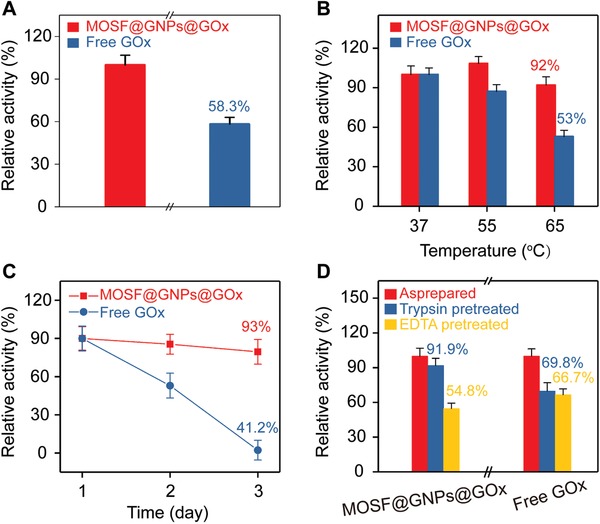
A) Catalytic activity examination for GOx before and after immobilization in MOSF. B) Temperature‐dependent activity comparison between MOSF@GNPs@GOx and free GOx. C) Long‐term stability comparison between MOSF@GNPs@GOx with free GOx. D) Catalytic activity comparison between MOSF@GNPs@GOx and free GOx against trypsin enzymolysis and EDTA denaturation.

We examined the enzyme activity by using glucose as a model substrate and 3‐MPBA‐modified GNPs as glucose indicators. MOSF@GNPs themselves exhibited no catalytic activity as evidenced by analyzing the SERS spectra of 3‐MPBA‐functionalized GNPs with and without glucose (Figure S9, Supporting Information). Compared to free GOx under the same conditions, the conversion of glucose by the MOSF@GNPs@GOx nanoreactors increased by ≈1.7 times (**Figure** **3**A). This was possibly due to the preconcentration of GOx in the MOSF nanopores and the decreased distance for the enzymatic byproduct H_2_O_2_ to diffuse to the boronate‐anchored gold particle surface.^[^ ^25^ ^]^ To evaluate enzyme thermal stability, we measured the oxidation extent of glucose under different reaction temperatures (Figure 3B). Both free GOx and MOSF‐confined GOx exhibited a high catalytic activity after incubation at 37 °C. However, the incubation of free GOx above 55 °C resulted in an obvious decline (up to 47% at 65 °C) of the conversion of glucose. In contrast, GOx confined in MOSF yielded around 92% conversion above 55 °C, indicating the stable catalytic activity under high temperature.

We further investigated the long‐term stability, by storing the free GOx solution and the MOSF‐confined GOx suspension for varied periods at room temperature (Figure 3C). After storage for 2 days, the conversion of MOSF‐encapsulated GOx remained stable (93% of the initial value), in comparison with dramatic decrease in free GOx (41% of the initial value). Except for the standards detection, the degradation by digestive enzymes and deactivation in the presence of chelating compounds are needed to be addressed in biological medias. For the effect of digestive enzymes, free GOx lost nearly half of the overall activity after being digested by trypsin for 30 min. In contrast, the activity of MOSF‐confined GOx reduced by less than 9% under the same condition (Figure 3D). This also implied that most enzyme molecules were adsorbed in the nanopores instead of on the surface of MOSF. For the effect of chelating compound, either free or immobilized GOx was found to lose >30% of its original activity in the presence of 1 wt% of EDTA. Although showing inadequate protection of enzyme from deactivation caused by chelating agents, the above result in turn verified that the MOSF allows small molecules to freely diffuse in and out of their nanopores, which is crucial for the detection of small molecule metabolites. Notably, the nanoreactor not only was stable for large‐scale clinical use, but also enabled downstream metabolite detection.

**Figure 4:**
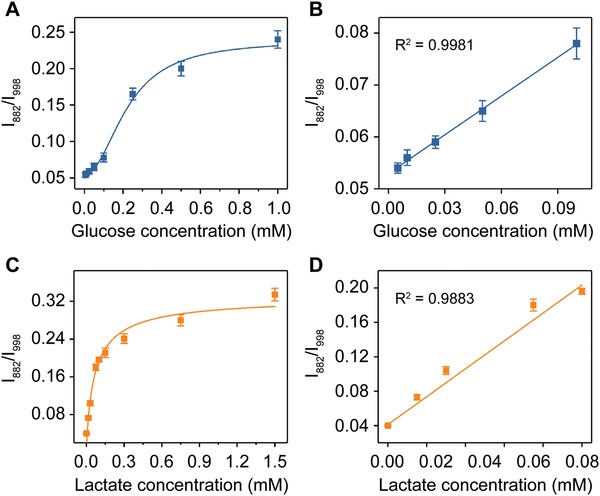
A) Plot of change in SERS intensity ratio (*I*_882_/*I*_998_) against glucose concentration. B) Linear fitting curve of SERS intensity ratio (*I*_882_/*I*_998_) from (A) against glucose concentration ranging from 5–100 × 10^−6^ m. C) Plot of change in SERS intensity ratio (*I*_882_/*I*_998_) against lactate concentration. D) Linear fitting curve of SERS intensity ratio (*I*_882_/*I*_998_) from (C) against lactate concentration ranging from 0–75 × 10^−6^ m.

The detection of target metabolites was achieved by using the nanoreactor as a smart SERS biosensor. For GOx‐loaded nanoreactor, as shown in **Figure** **4**A, the SERS intensity ratio of *I*_882_/*I*_998_ increased with the increase of glucose concentrations, and a linear range from 5 to 100 × 10^−6^ m was obtained (Figure 4B). Similarly, for the nanoreactor incorporating LOx, the relative SERS intensity increased as a function of the lactate concentration from 0 to 1.5 × 10^−3^ m, and a linear range from 0 to 75 × 10^−6^ m was obtained (Figure 4C,D). We collected SERS spectra from ten different locations of the glass capillary filled with the nanoreactor, and demonstrated the spectra reproducibility with the relative deviation of 5.2% (before glucose addition) and 7.5% (after glucose addition, Figure S10, Supporting Information). We obtained similar results for three batches of SERS detection in parallel, with an inter‐sample deviation of 3.5% (Figure S11, Supporting Information). In addition, no significant difference was observed in SERS responses before and after 24 h storage (Figure S12, Supporting Information). These results suggested that the biomimetic plasmonic nanoreactors may serve as a biosensing device for monitoring glucose toward clinical use with good reproducibility and stability.

**Figure 5:**
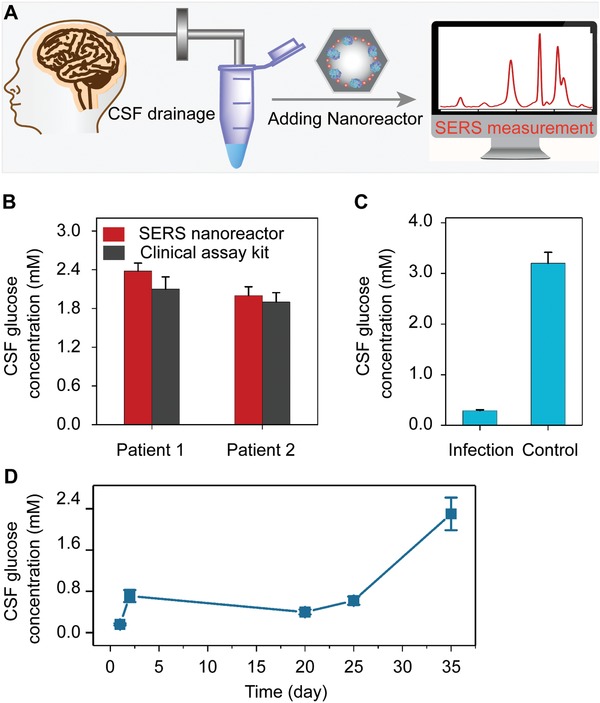
A) Scheme of monitoring glucose in patient CSF with plasmonic nanoreactor. B) Glucose concentrations in patient CSF determined with nanoreactor and clinical test kit. C) glucose concentrations of brain infection patient and noninfection control measured with plasmonic nanoreactor. D) Dynamic monitoring of CSF glucose of a brain infection patient during anti‐infectious therapy.

To challenge the plasmonic nanoreactor for rapid and reliable monitoring disease‐related metabolites in vitro, we analyzed glucose in CSF samples of patients suffering from bacterial CNS infection after neurosurgical operation. As one of the most common complications in neurosurgery, postoperative brain infection occurs at a rate of 0.5% to 18% depending on the specific surgical site and leads to prolonged postoperative hospital stay, increased treatment cost and risks of disability and mortality.^[^ ^26^ ^]^ The CSF glucose level has long been recognized as an important biochemical indicator for bacterial brain infection. We quantitatively measured the concentration of glucose in patient CSF (20 µL) without tedious pretreatment procedures (**Figure** **5**A). The glucose levels from two patient samples were 2.3 and 2.1 × 10^−3^ m, respectively, which were in agreement with the results of the clinical glucose assay kit (Figure 5B). The accuracy could be ascribed both to the high specificity of the enzyme toward its substrate molecule and to our nanoporous silica foams that isolated the enzymes from the external environments. Notably, we excluded the interference of the basal H_2_O_2_ in brain since its concentration is 2 to 4 orders of magnitude less than that of brain glucose.^[^ ^27^ ^]^ The infected patient shows a nearly tenfold decrease in the brain glucose in comparison with uninfected control (Figure 5C), due to the reduced uptake of oxygen and glucose into brain and the increased glycolysis by postoperative bacteria.^[^ ^1^, ^28^ ^]^ We further applied the nanoreactors to monitor the dynamics of the patient CSF glucose during anti‐infection treatment. As shown in Figure 5D, after receiving continuous antibiotic treatment, the patient's CSF glucose level gradually increased from the initial value of 0.1 × 10^−3^ m to the 2.38 × 10^−3^ m after 35 days of therapy. Notably, current clinical testing of CSF glucose is limited, concerning instability of the enzyme, low sensitivity of the conventional colorimetric readout, and large sample consumption (≈200 µL).^[^ ^29^ ^]^ For comparison, our approach offers enhanced analytical stability (at least 24 h) and reduced sample consumption (20 µL) by approximately tenfold decrease. We demonstrated the potential applicability of our SERS nanoreactor for in vitro metabolic diagnosis of postoperative brain infection and monitoring of clinical therapeutic efficiency.

## 3 Conclusion

In summary, we have, for the first time, reported the design and fabrication of a porous silica‐based SERS nanoreactor for reliable metabolic analysis in biofluids. With the advantages of large specific surface area, good stability, light transparency, and easy operation of MOSF, the nanoreactor is capable of immobilizing large amounts of enzyme molecules and plasmonic nanoparticles in spatially confined nanopores. The nanoreactor shows improved enzyme catalytic activity and stability against external conditions including high temperature, protease digestion, and long‐term storage. On the other hand, the integration of SERS enhancers and ratiometric Raman reporters into the biocatalytic microenvironment enables the nanoreactor to quantify the physiological and pathological concentration of target metabolite, as exemplified by the detection and monitoring of CSF glucose from patients suffering from postoperative bacterial CNS infection. Although the present work focus on the use of GOx/LOx and spherical GNPs as model enzymes and SERS substrates, we believe that this porous silica‐templated assembly strategy can be extended to encapsulate different enzymes, Raman enhancers, and reporters within the nanopores for analysis of various disease‐related metabolites (e.g., uric acid, cholesterol, and amino acids) in biofluids. We foresee the development of point‐of‐care and bedside in vitro clinical testing based on these nanoreactors, enhancing the diagnostic performance and benefiting precision medicines in the near future. The development of multi‐enzyme cascade SERS nanoreactors with extended applicability and further improved sensitivity is ongoing in our laboratory.

## 4 Experimental Section

##### Reagents

Poly (ethylene oxide)‐poly (propylene oxide)‐poly (ethylene oxide) (EO20PO70EO20) copolymer (typical molecular weight (MW) ≈5800, denoted as P123), TMOS (98%), and lactate acid (≥98%) were purchased from Sigma Aldrich (Shanghai, China). Sodium sulfate anhydrous (≥99%) was purchased from Shanghai Dahe Chemical Co., Ltd. (Shanghai, China). Tetrachloroauric acid tetrahydrate (HAuCl_4_·4H_2_O, Au ≥47.8%), citrate trisodium dihydrate (≥99%), 3‐mercaptophenylboronic acid (3‐MPBA, ≥95%), 3‐hydroxythiophenol (3‐HTP, ≥98%), and glucose (≥99%) were purchased from Sinopharm Chemical Reagent (Shanghai, China). Glucose oxidase (GOx) and lactate oxidase (LOx) were purchased from Shanghai Yuanye Bio‐Technology Co., Ltd. (Shanghai, China). Other chemicals were of at least analytical reagent grade and used as received. Deionized water (18 MΩ cm) used throughout the experiments was obtained from a Milli‐Q system (Millipore, Bedford, MA, USA).

##### Apparatus

Transmission electron microscopy analysis was performed on a Tecnai G2 20 TWIN transmission electron microscope operated at an acceleration voltage of 200 kV (FEI, USA). Scanning electron microscopy analysis was performed on a Zeiss Ultra 55 scanning electron microscope operated at an acceleration voltage of 5 kV (Carl Zeiss Microscopy, Germany). Nitrogen sorption analysis was performed at −196 °C using a Quadrasorb SI analyzer from Quantachrome Instrument (Boynton Beach, USA). The samples were degassed at 250 °C in vacuum for at least 3 h prior to analysis. UV–visible (UV–vis) extinction spectra were measured on a UV8000 UV–vis spectrophotometer with a 1.0 cm quartz cell (Shanghai Metash Instruments Co. Ltd., Shanghai, China). Raman and SERS spectra were determined on a Horiba XploRA confocal Raman microscope (Jobin Yvon, France) after loading samples into glass capillaries (inner diameter = 500 µm). A 638 nm diode laser was used to excite the SERS emission through a 10 × Olympus objective (NA = 0.25). The integration time for each spectrum was 10 s, and five different locations were determined for each sample. The narrow peak of monocrystalline Si at 520 cm^−1^ was used to correct the system before analysis. Prior to extraction of the peak intensities, all spectra were subjected to baseline correction and smoothing with the LabSpec 6 software (Jobin Yvon, Horiba Gr, France).

##### Synthesis of MOSF

MOSF was synthesized according to the previous reports.^[^ ^15^, ^16^ ^]^ Briefly, 1.3 g of P123 was stirred with 1.7 g of sodium sulfate anhydrous in 200 × 10^−3^ m of HAc‐NaAc buffer solution (pH = 5.0) at room temperature for 10 h. The solution was then heated to 40 °C, followed by the addition of 1.48 mL of TMOS as the silica source under stirring. Afterward, the mixed solution was allowed to stand for 24 h. The crude product was subsequently subjected to hydrothermal treatment at 100 °C for 24 h. Finally, the purified MOSF products were baked at 550 °C for 5 h to remove the template.

##### Synthesis of GNPs

GNPs with different sizes were synthesized via the classical Turkevich‐Frens method with minor modification.^[^ ^30^ ^]^ Briefly, 50 mL of HAuCl_4_ solution (0.01 wt%) was heated to boiling, followed by the addition of 0.65 mL of trisodium citrate solution (1 wt%) under vigorous stirring. The solution was continually boiled for 30 min to ensure the complete reduction of HAuCl_4_. The resulting particles were about 30 nm in diameter according to SEM characterization. For 20 and 60 nm GNPs, the volume of citrate was changed to 1 and 0.4 mL, respectively, while keeping other procedures unchanged. The concentrations of three types of GNPs were determined through UV–vis spectroscopy using different molar extinction coefficients, including 1.03 × 10^9^ (for particles of 20 nm), 3.96 × 10^9^ (for particles of 30 nm), and 3.06 × 10^10^ m ^−1^ cm^−1^ (for particles of 60 nm).^[^ ^31^ ^]^ For each nanoparticles, the concentration was adjusted to ≈0.1 × 10^−9^ m with deionized water before further use.

##### Preparation of Plasmonic Nanoreactors

1 mg of MOSF was dispersed in 1 mL of GNP solution, followed by continuous shaking at 25 °C for 4 h to achieve adsorption equilibrium. The silica nanofoams were subjected to centrifugation (3500 rpm, 5 min) for three times and re‐dispersed in 1 mL of water. Then, 16 × 10^−6^ m of 3‐MPBA ethanol solution (1 × 10^−3^ m) was added in the silica dispersion with vigorous shaking. After 1 h incubation, the silica nanofoams were subjected to centrifugation (3500 rpm, 5 min) in water for three times to remove the free boronic acid. The nanofoams were dispersed in 500 µL of enzyme solution and stirred at 25 °C for 2 min, followed by centrifugation (3500 rpm, 7 min) in water for three times. Finally, the as‐prepared nanoreactors were re‐dispersed in 100 µL of phosphate buffer solution (pH 7.4, 10 × 10^−3^ m) for further use.

##### Evaluation of Enzyme Stability

To evaluate the catalytic activity before and after immobilization, 100 µL of plasmonic nanoreactors and 100 µL of 3‐MPBA‐GNPs (1 × 10^−9^ m) containing 45 µg of GOx were mixed with 10 µL of glucose (5 × 10^−3^ m), respectively. The above mixtures were incubated at 37 °C for 30 min. For thermal stability, free GOx and plasmonic nanoreactors were mixed with 10 µL of glucose (5 × 10^−3^ m), followed by incubation at different temperature (e.g., 37, 55, and 65 °C) for 30 min. For the long‐term stability, free GOx and nanoreactors were stored at room temperature for different time period (e.g., 1 day, 2 days, and 3 days) before incubation with glucose. For activity influenced by trypsin or EDTA, free GOx and plasmonic nanoreactors were pretreated with 10 × 10^−6^ m of trypsin or 1 wt% EDTA solution for 30 min before incubation. The stability of above samples was measured in terms of relative conversion of 3‐MPBA oxidation as described previously.^[^ ^6^, ^18^ ^]^

##### SERS Detection of Metabolites with Nanoreactors

80 µL of nanoreactors was incubated with 20 µL of glucose or lactate solution at 37 °C for 30 min. The SERS spectra were collected following the previous protocols in standard analysis.

##### SERS Detection of Metabolites in Patients' CSFs

The CSF samples were donated by patients from Shanghai Children's Hospital. The investigation protocol was approved by the institutional ethics committee of Shanghai Children's Hospital. Informed consent was obtained from all sample donors. All samples were collected by continued postoperative drainage and stored at −20 °C before analysis to prevent sample degradation. 80 µL of nanoreactors containing GOx was incubated with 20 µL of CSF at 37 °C for 30 min. The SERS spectra were collected following the previous protocols in standard analysis.

## Conflict of Interest

The authors declare no conflict of interest.

## Supporting information

Supporting Information
