# Supplementary material for: A Biomimetic Plasmonic Nanoreactor for Reliable Metabolite Detection
Source: Adv Sci (Weinh). 2020 Mar 11;7(10):1903730. doi: 10.1002/advs.201903730 (PMC7237842; doi:10.1002/advs.201903730)
Supplement: Supplementary file 1 — Supporting Information [file ADVS-7-1903730-s001.pdf]

Copyright WILEY-VCH Verlag GmbH & Co. KGaA, 69469 Weinheim, Germany, 2020.

ADVANCED SCIENCE
Open Access

# Supporting Information

for *Adv. Sci.,* DOI: 10.1002/advs.201903730

## A Biomimetic Plasmonic Nanoreactor for Reliable Metabolite Detection

*Jiangang Liu, Chenlei Cai, Yuning Wang, Yu Liu, Lin Huang, Tongtong Tian, Yuanyuan Yao, Jia Wei, Ruoping Chen,* Kun Zhang,* Baohong Liu,* and Kun Qian**

# Supporting Information

## A Biomimetic Plasmonic Nanoreactor for Reliable Metabolite Detection

*Jiangang Liu,*[†,#] *Chenlei Cai,*[‡,#] *Yuning Wang,*[§] *Yu Liu,*[†] *Lin Huang,*[†] *Tongtong Tian,*[§] *Yuanyuan Yao,*[§] *Jia Wei,*[†] *Ruoping Chen,*[*,†] *Kun Zhang,*[*,†] *Baohong Liu,*[*,§] *and Kun Qian*[*,†]

## Table of Contents

**Figure S1.** Microscopic characterization of MOSF. ........................................................ 2

**Figure S2. N**itrogen adsorption analysis of MOSF. ........................................................ 3

**Figure S3.** Extinction of MOSF@GNPs and supernatants after repeated centrifugation. ........... 4

**Figure S4.** Optimization of GNPs immobilization time. ................................................ 5

**Figure S5.** Optimization of GNPs concentration. ........................................................ 6

**Figure S6.** Optimization of 3-MPBA solution volume. ................................................ 7

**Figure S7.** Optimization of 3-MPBA modification time. ................................................ 8

**Figure S8.** UV/vis absorption spectra of (i) the original glucose oxidase solution and (ii) the supernatant after immobilization onto MOSF@GNP. .................................. 9

**Figure S9.** SERS spectra of MOSF@GNPs in presence and absence of glucose. .................... 10

**Figure S10.** Intra-sample reproducibility test. .......................................................... 11

**Figure S11.** Inter-sample reproducibility test. .......................................................... 12

**Figure S12.** Time-dependent stability of SERS test. ...................................................... 13

**Table S1.** Detection performance comparison between different SERS sensors for $H_2O_2$. .......... 14

**Supplementary References.** ....................................................................... 15

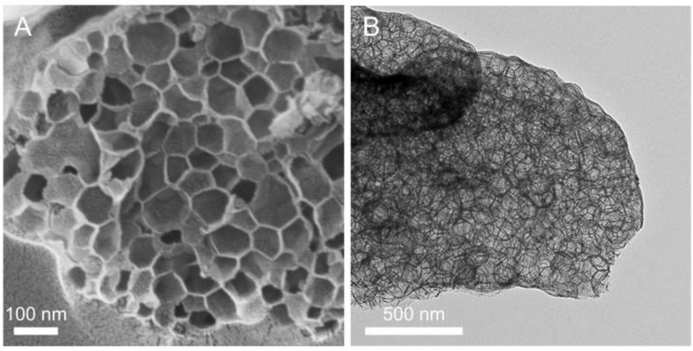


**Figure S1.** Microscopic characterization of MOSF. (A) SEM and (B) TEM images of MOSF. The porous silica material displays a typical foam-like structure with a pore size of ~75 nm and a wall thickness of ca. 3~5 nm.

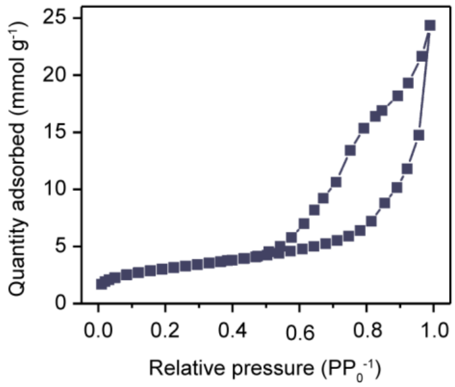


**Figure S2.** Nitrogen adsorption analysis of MOSF. MOSF displays a type IV isotherm with an H1 hysteresis loop in high relative pressure ($PP_0^{-1}$) range.

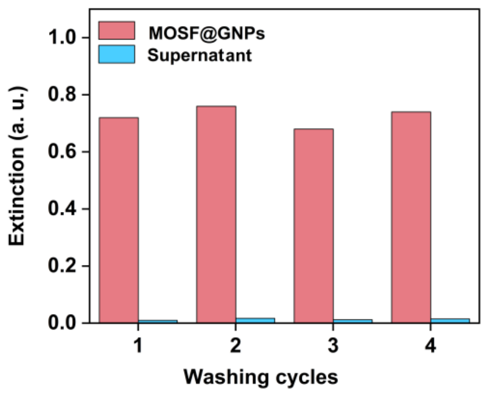


**Figure S3.** Extinction of MOSF@GNPs and supernatants after repeated centrifugation of MOSF@GNPs.

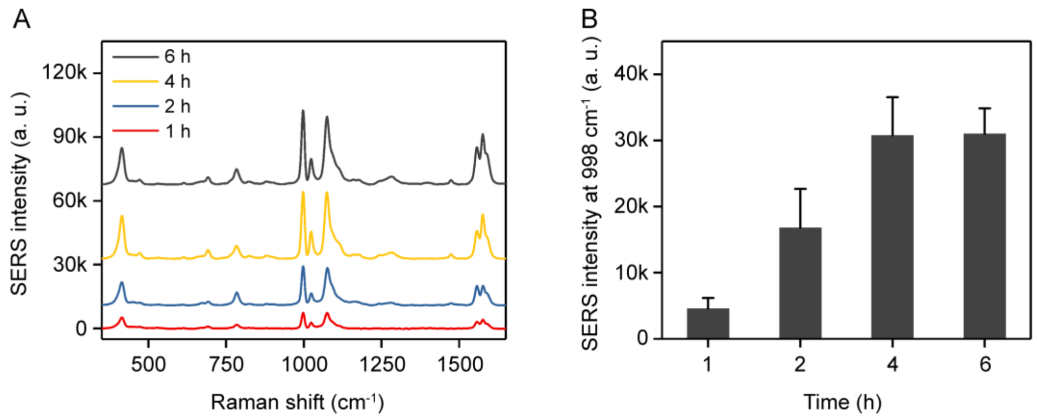


**Figure S4.** Optimization of GNPs immobilization time. (A) SERS spectra obtained from MOSF@GNPs after interaction with GNPs (30 nm) for different times. (B) Bar chart showing change in peak intensity of 3-MPBA at 998 $cm^{-1}$ against adsorption time of GNPs.

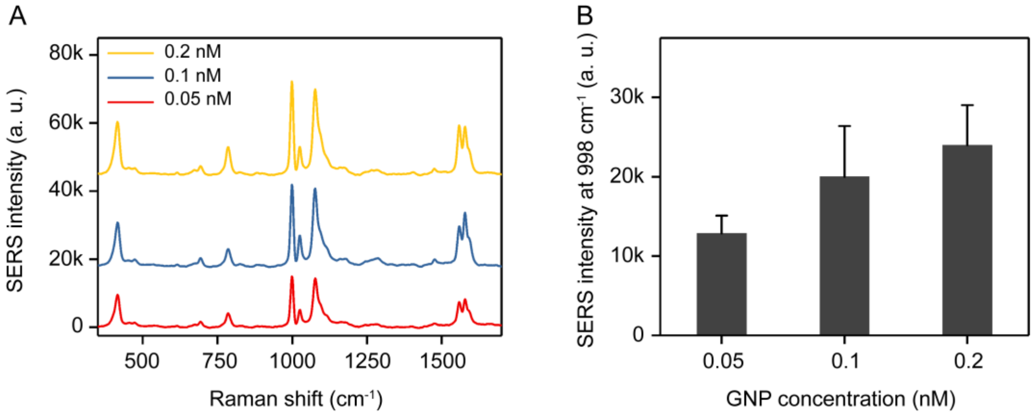


**Figure S5.** Optimization of GNPs concentration. (A) SERS spectra obtained from MOSF@GNPs after reaction with different concentrations of GNPs (30 nm). (B) Bar chart showing change in peak intensity of 3-MPBA at 998 $cm^{-1}$ against concentration of GNP solution.

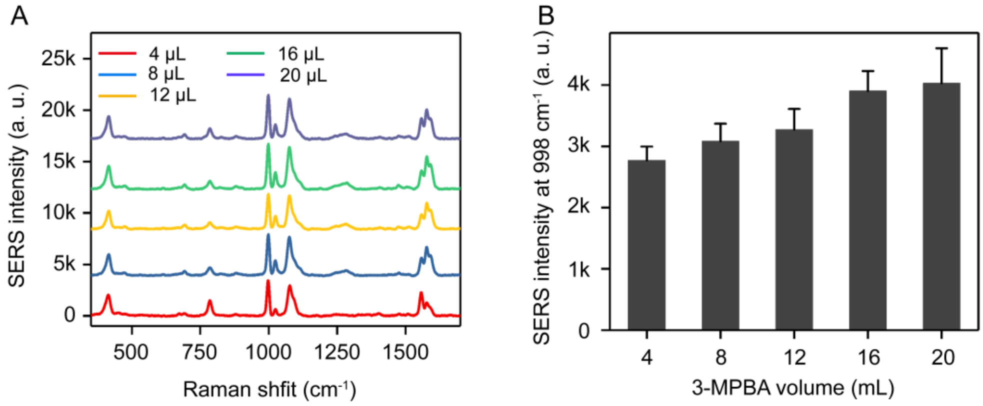


**Figure S6.** Optimization of 3-MPBA solution volume. (A) SERS spectra obtained from MOSF@GNPs after reaction with different amounts of 3-MPBA (1 mM). (B) Bar chart showing change in peak intensity of 3-MPBA at 998 $cm^{-1}$ against volume of 3-MPBA solution.

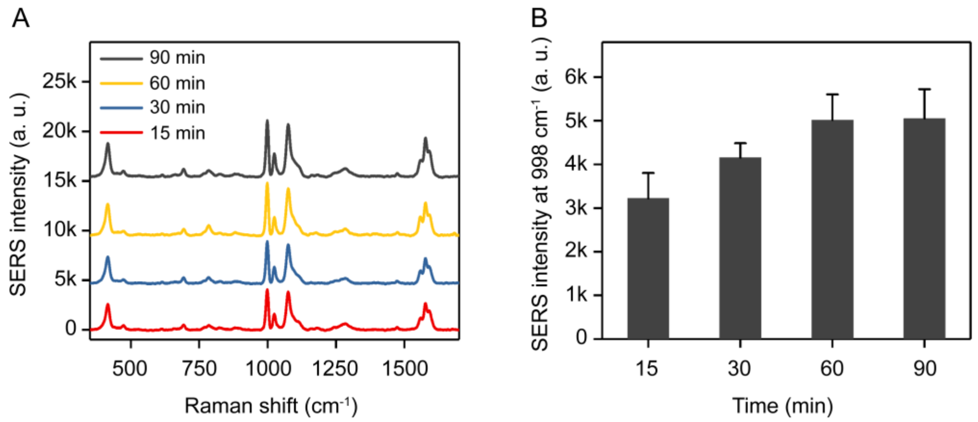


**Figure S7.** Optimization of 3-MPBA modification time. (A) SERS spectra of 3-MPBA adsorbed on MOSF@GNPs with different modification times. (B) Bar chart showing change in peak intensity of 3-MPBA at 998 $cm^{-1}$ against modification time.

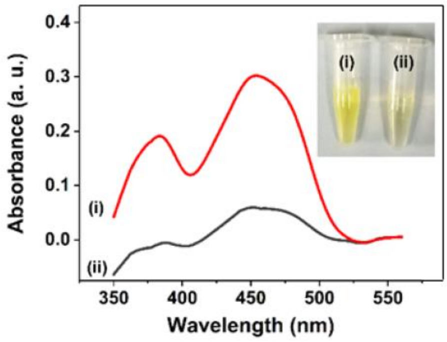


**Figure S8.** UV/vis absorption spectra of (i) original glucose oxidase solution and (ii) supernatant after immobilization onto MOSF@GNP. Insert shows corresponding digital graphs of GOx solutions before and after immobilization.

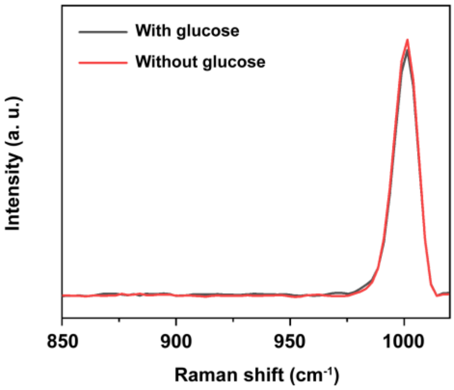


**Figure S9**. SERS spectra of MOSF@GNPs in presence and absence of glucose.

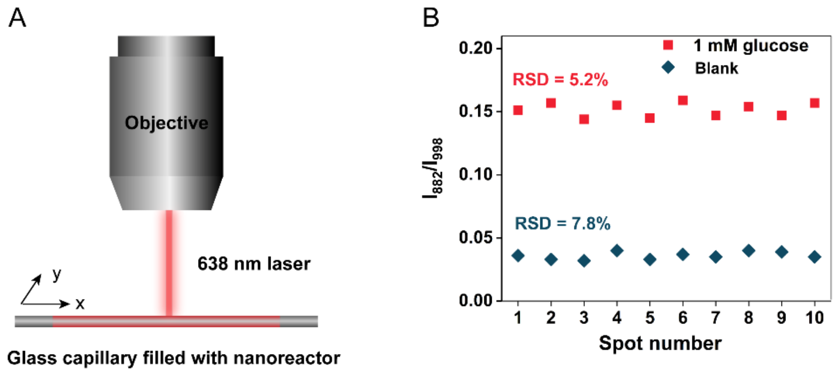


**Figure S10.** Intra-sample reproducibility test. (A) Scheme of recording SERS spectra from different locations of capillary filled with nanoreactor. (B) Deviation of relative SERS intensities measured from 10 spots as shown in (A).

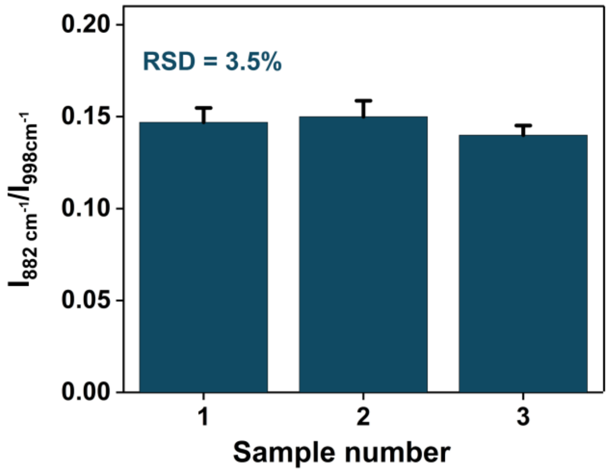


**Figure S11.** Inter-sample reproducibility test.

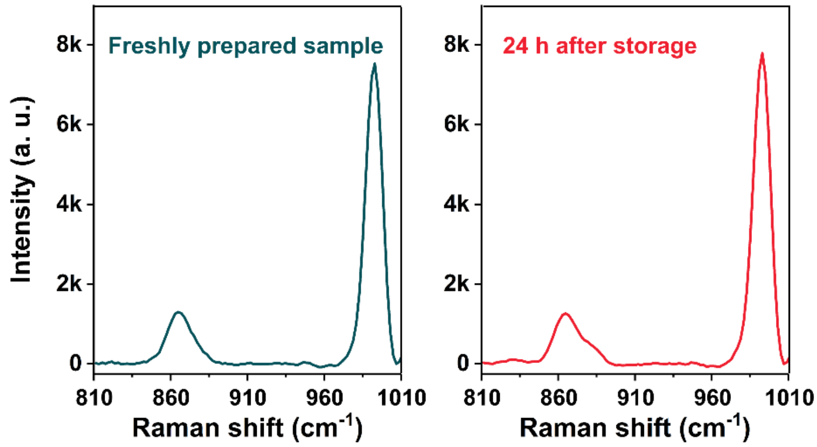


**Figure S12.** Time-dependent signal stability test.

**Table S1.** Detection performance comparison between different SERS sensors for $H_2O_2$

| Probe | Dynamic range (μM) | LOD (μM) | Ref. |
|---|---|---|---|
| Gold nanorods | 1~100 | 0.3 | S1 |
| Gold nanoparticle aggregates | 0.1~2.53 | 0.07 | S2 |
| Ag@Au core/shell nanoparticles | 0~300 | 0.45 | S3 |
| Porous $SiO_2$-coated Au-Ag alloy nanoparticles | 0.12~8 | 0.052 | S4 |
| Gold nanoparticle array | 0.05~1 | 5.5 | S5 |
| Gap-mode Au@Ag core/shell nanoparticles | 0~100 | 5.5 | S6 |
| MOSF@GNPs | 1~100 | 0.47 | This work |

**Supplementary References**


S1. R. Peng, Y. Si, T. Deng, J. Zheng, J. Li, R. Yang, W. Tan, *Chem. Commun.* **2016**, *52*, 8553.

S2. X. Gu, H. Wang, Z. D. Schultz, J. P. Camden, *Anal. Chem.* **2016**, *88*, 7191.

S3. L. Bai, X. Wang, K. Zhang, X. Tan, Y. Zhang, W. Xie, *Chem. Commun.* **2019**, *55*, 12996.

S4. Y. Si, L. Li, X. Qin, Y. Bai, J. Li, Y. Yin, *Anal. Chim. Acta.* **2019**, *1057*, 1.

S5. K. Zhang, Y. Wang, M. Wu, Y. Liu, D. Shi, B. Liu, *Chem. Sci.* **2018**, *9*, 8089.

S6. X. Jiang, Z. Tan, L. Lin, J. He, C. He, B. D. Thackray, Y. Zhang, J. Ye, *Small Methods* **2018,** *2*, 1800182.
